# Korean medicine registry of herbal medicine for weight loss

**DOI:** 10.1097/MD.0000000000029407

**Published:** 2022-06-10

**Authors:** Mi Mi Ko, Bo-Young Kim, Mi Ju Son, Kyung Hwan Jegal, Won-Seok Chung, Sungha Kim

**Affiliations:** a KM Science Research Division, Korea Institute of Oriental Medicine, Yuseong-gu, Daejeon, Republic of Korea; b College of Korean Medicine, Daegu Haany University, Gyeongsan, Republic of Korea; c Department of Clinical Korean Medicine, Graduate School, Kyung Hee University, Seoul, Republic of Korea.

**Keywords:** herbal medicine, Korean medicine, obesity, prospective observational study, registry, weight loss, real-world data

## Abstract

**Introduction:**

In South Korea, the prevalence of obesity has continuously increased over the last decade, and the burden of social and economic costs has also increased immensely. The purpose of this study is to investigate the clinical characteristics and current status of patients receiving herbal medicine (HM) treatment for weight loss in Korean medicine (KM) by constructing a multi-institutional prospective registry.

**Methods and analysis:**

This is a prospective, observational, multi-center registry, including patients visiting the KM clinics in South Korea for weight loss. This study will collaborate with 15 KM clinics and recruit patients into the registry between October 2021 and October 2022. The study population will include patients visiting the KM clinics for weight loss. A total of 1000 eligible patients visiting the KM clinics for weight loss who decide to undergo HM treatment will be enrolled in the registry. After enrollment, we will collect the individual characteristics of each patient, such as body mass index, body composition test, liver and kidney function tests, and information on prescribed HM. We will also record the adverse events at each visit.

**Discussion:**

This study is the first prospective, multicenter registry of HM for weight loss in KM clinics. The results of this registry may show the current status of patients who receive HM treatments for weight loss and provide evidence for reasonable decision-making on KM healthcare policy for obese patients in the future.

## 1 Introduction

Obesity is a major risk factor of various diseases, such as cardiovascular diseases and metabolic diseases. The prevalence of obesity and overweight has been increasing gradually over the past decades. Consequently, the burden of social and economic costs has also been increasing immensely. In South Korea, the prevalence of obesity continuously increased from 29.7% in 2009 to 35.7% in 2018 in the total population.^[1]^ The total social and economic losses for obesity in Korea was 11.5 trillion KRW (10 billion USD) in 2016, which represents 0.7% of the Korean gross domestic product, and approximately 50% of the costs were medical expenses.^[2]^ With the increase in obese population, attempts to lose weight have also been continuously increasing with various weight control methods, such as regular physical activities, diet control, such as calorie restriction, and frequent weight monitoring.^[3]^ However, even if weight loss is achieved, its long-term results are generally poor and sustaining reduced weight can be difficult with lifestyle changes alone, and additional medical therapies may be needed.^[4]^ Among the weight control methods, herbal medicine (HM) is one of the most effective strategies in the Korean population.^[5,6]^

Korean medicine (KM) is recognized as the double axis of health care system of South Korea along with conventional western medicine. Supportive evidence has demonstrated the safety and effectiveness of HM for obesity compared with conventional medicine.^[7–11]^ However, it is also reported that some herbal formulas are associated with several adverse events (AEs),^[12,13]^ and in some cases, the government has imposed restrictions on the use of certain medicines because of safety concerns.^[14]^ In Korea, because of the low insurance coverage for KM obesity treatments, including prescribed HM, information, such as the prescribed medication and its AEs in real clinical practice of KM treatment for obesity are scarce. As part of the real clinical practice of KM treatment for obesity, many studies on KM registry are currently being attempted in KM.^[15–18]^ This registry study was designed to understand the characteristics of KM weight loss treatment, which is commonly used in Korea, centered on primary medical institutions for the first time in the KM field.

The purpose of this study is to investigate the clinical characteristics and current status of individuals who receive HM for weight loss in KM by constructing multi-institutional prospective registry. Overall, the aims of this registry are to:

(1) investigate the clinical characteristics and current status of treatment for weight loss;
(2) evaluate the safety of various HMs for weight loss, based on nationwide registry outcome collecting process.

## 2 Participants and methods

### 2.1 Study design/setting

**Figure 1:**
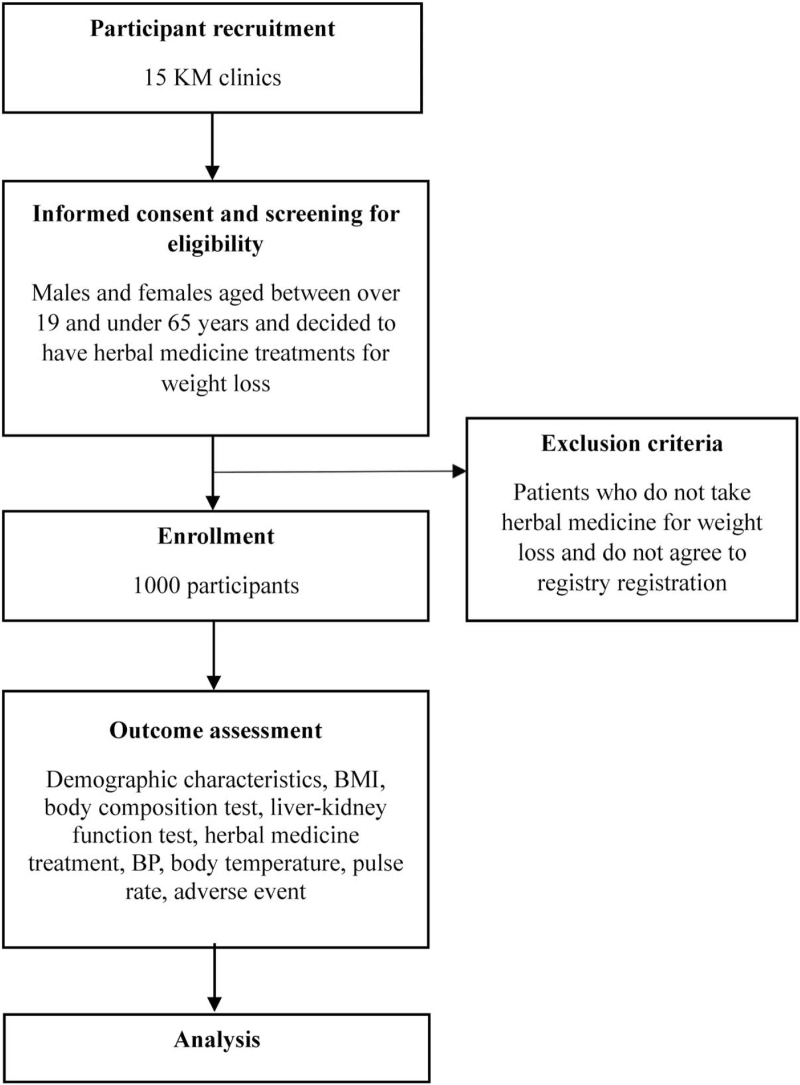
Study flow chart. BMI = body mass index, BP = blood pressure, KM = Korean medicine.

This is a prospective observational multi-center study enrolling patients undergoing KM weight loss treatment in Korea. We will recruit patients from the following 15 KM clinics: Bareun-mom S KM clinic (Hwaseong, Gyeonggi-do), Saeng-saesang KM clinic (Daejeon), Sangju Baraeun KM clinic (Sangju, Gyeongsang-do), Hwapyeong KM clinic (Incheon), Imom KM clinic (Changwon, Gyeongsang-do), Goeun KM clinic (Gunpo, Gyeonggi-do), Lee Seung Jin KM clinic (Namyangju, Gyeonggi-do), Onki KM clinic (Namyangju, Gyeonggi-do), Chunjin KM clinic (Boryeong, Chungcheon-do), Sogood KM clinic (Seongnam, Gyeonggi-do), Gyeongsan S KM clinic (Gyeongsan, Gyeongsang-do), Myoungjak KM clinic (Gunsan, Jeolla-do), Momandjang KM clinic (Gunpo, Gyeonggi-do), Kyurim KM clinic (Cheongju, Chungcheon-do), and Madimadi KM clinic (Yongin, Gyeonggi-do) between October 2021 and October 2022 (Fig. 1). Patients who provide informed consent to participate will receive KM weight loss treatment according to their individual characteristics without any intended intervention. At baseline (recruitment), comprehensive data will be collected on the following variables: demographics, anthropometric information, medical history, laboratory tests, treatment, and AEs. An electronic care report form (eCRF) was developed using the myTrial system^[19]^ of the National Agency for Development of Innovative Technologies in Korean Medicine (IT-KgoM), which supports public eCRFs. Table 1 shows the data collection and follow-up schedules.

**Table 1:** Study schedule of the registry study.

	Enrollment baseline	Subsequent visits	End of treatment
Informed consent	V		
Eligibility screening	V		
Demographic characteristics	V		
Body mass index	V		V
Body composition test	V		V
Liver function tests	V		V
Kidney function tests	V		V
Herbal medicine	V	V	V
Blood pressure, body temperature, pulse rate	V		V
Adverse event		V	V

### 2.2 Study registration

This study is registered with the Clinical Research Information Service (https://cris.nih.go.kr/cris/en/): KCT0006792. Current protocol versions are 1.2.

### 2.3 Eligibility criteria

#### 2.3.1 Inclusion criteria

We will include eligible patients according to the following criteria:

(1) Men and women aged between 19 and under 65 years of age.
(2) Among the patients who visited the hospital for weight loss, those who decided to undergo HM treatment.
(3) Patients who have listened to sufficient explanation before registry registration, agreed to participate in this study voluntarily, and signed written informed consent approved by the institutional review board.

#### 2.3.2 Exclusion criteria

(1) Patients who do not take HM for weight loss.
(2) Patients who do not agree to registry registration.
(3) Participants who are judged to be inappropriate for participating in this study.

### 2.4 Recruitment

The participants will be recruited from KM obesity clinics of 15 KM primary medical institutions across Korea. The researcher will fully explain the aim of this study and details of the procedures, including research usage, confidentiality, and providing data provision to third parties for analysis and will obtain informed consent from the potential participants who met the inclusion criteria before the collection of information.

Participants will be free to withdraw at any time during the study, and this will not affect their clinical treatment. We encourage voluntary participation in this study by posting recruitment posters on the walls of the entrance and elevators of the each clinic.

### 2.5 Exposure

The purpose of this study is to observe various situations from routine clinical practice, which differs from other clinical trials that evaluate the efficacy of specific interventions.

The participants will receive individualized general HM in KM treatments. There will be no added interventions for this study, and general KM treatments will be administered to the patients in accordance with safety regulations. The KM doctors, who are participating voluntarily, have over 5 years of clinical experience treating patients with obesity.

### 2.6 Outcomes measures

Researchers determined that the following data will be collected for observing the characteristics of KM weight loss treatment. Detailed research schedule and variables are presented in Table 2.

(1) *Demographics*: Age, sex, and medical history will be collected as basic demographic information.
(2) *Anthropometric data*: If a participant visits physically for consultation, vital signs, including blood pressure, body temperature, and pulse rate will be collected as variables related to general health conditions. Weight, height, and body mass index (BMI) will be measured as variables for weight change. Body fat mass, body muscle, visceral fat level, etc will be measured as variables for changes in body composition.
(3) *Liver and kidney function tests*: To evaluate the physical changes induced by long-term administration of HM or weight loss, liver function (aspartate aminotransferase, alanine aminotransferease) and renal function tests (blood urea nitrogen, creatinine) will be conducted at the visit.
(4) *Other laboratory test*: Total cholesterol, high-density lipoprotein, low-density lipoprotein, triglycerides, etc will be evaluated at the visit to evaluate the physical changes induced by long-term administration of HM for weight loss.
(5) *HM treatment*: For the observation and collection of real clinical data, there will be no restriction on the type or frequency of HM treatment, and doctors in the primary KM clinics will perform the HM treatment according to the characteristics of the individuals. For a given period, prescription medication will be recorded with the name of the medicine, medicine composition, and treatment duration.
(6) *AEs*: At each treatment visit, participants will be investigated by the KM doctors using the AEs template developed by the expert Delphi group to evaluate the occurrence of any AE. In case of occurrence of any AE, we will immediately provide appropriate treatment to the participant according to the guidelines of the clinic, and then observe the participant's progress during the follow-up visit. The name, severity, and duration of the AEs that occur during the study period will be recorded. In addition, any causality assessments between the AE and the KM treatment will be evaluated by the investigator during visits. All investigators and KM doctors will be educated in research ethics (Table 2).

**Table 2:**
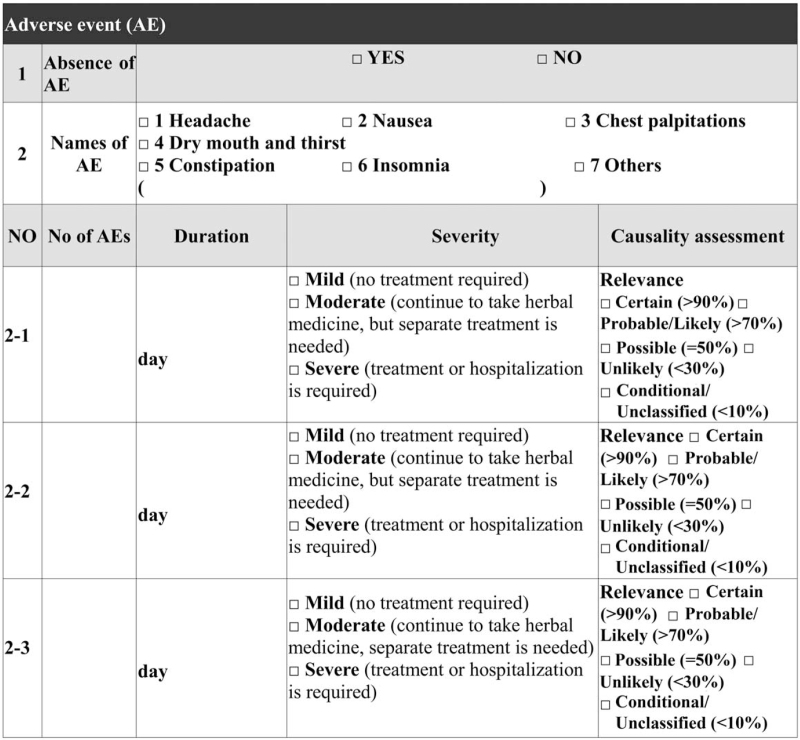
Template of adverse events of herbal medicine.

### 2.7 Data management and quality control

The data collection uses the eCRF system (myTrial electronic data capture) verified by IT-KoM. The KM doctors in charge of each clinic will check the clinical measurement results of the participant assessed at each visit, and then, will input and save them into the eCRF system. To access a data entry form, each KM doctor on a remote computer with an internet connection enters the address of the web server (http://ecrf.nikom.or.kr). Then, a login screen is displayed and the each user enters a user identification and password, provided by personnel responsible for the web site. Each case entered into the registry will be periodically reviewed, and data queries will be generated to provide feedback to the KM clinics by a clinical research associate, based on the data monitoring manual. Data for this study will be stored safely in a dedicated collection server and this server will grant limited data access rights.

### 2.8 Sample size calculation

We plan to recruit at least 1000 patients during this study period. Empirically considering the trends of the patients visiting each primary KM clinics, we estimated that approximately 1000 participants will be enrolled (65 patients or more per year per clinic) for a year. The study started in October 2021, is currently ongoing, and will finish in October 2022.

### 2.9 Statistical analysis

All statistical analyses will be conducted via two-tailed tests, and the significance level will be set at 5%. Categorical variables will be presented as n (%) whereas continuous variables will be expressed as mean ± standard deviation. For comparison of two or more groups, categorical variables will be subjected to the chi-square test and Fisher's exact test, whereas continuous variables will be analyzed using the *t* test and analysis of variance or non-parametric methods, such as Mann–Whitney *U* test and Kruskal–Wallis test, for those not meeting the normality criteria. Pre- and post-treatment comparison conducted with a paired *t* test or Wilcoxon signed rank test will be analyzed using a Kolmogorov–Smirnov test. Analysis of covariance will be performed when it is necessary to control the underlying variables, such as demographic variables. For data with a missing value, the last observation carried forward will be used through case report form (CRF) inspection to replace the missing value. All statistical analyses in this study will be conducted using SAS software, version 9.4 (SAS Institute, Inc., Cary, NC).

### 2.10 Ethics and dissemination

The IRB of the Kyung Hee University, Seoul, Republic of Korea (KHSIRB-21-447(RA)) approved the study. Written informed consent will be obtained from all study participants before enrollment in the study. The results will be published in a peer-reviewed journal and disseminated electronically and in print regardless of the results.

## 3 Discussion

This paper describes the protocol of a prospective, multicenter, observational registry study to monitor the changes in symptoms and safety of patients who have been treated with HM in Korea. In evidence-based medicine, the number of systematic observational studies actually performed in this field is very small, although the appropriateness of clinical observational studies is often emphasized. A carefully planned and performed prospective observational study does not guarantee an outcome showing a definitive relationship between therapeutic intervention and efficacy and safety, and there are clearly limits to the research design itself for the generalization of the results.

In recent years, in the era of big data, the demand for the use of medical big data is increasing. Especially, with the development of artificial intelligence, the use of large-scale medical data and the necessity of individualized customized treatments are being highlighted. Therefore, many researchers are interested in structuring and standardizing data to properly use electronic medical record (EMR) clinically and are trying to develop guidelines or certification standards for its use.^[20]^ However, in KM, researchers face several obstacles in utilizing EMR information of KM because of the difficulty in patient follow-up, non-reimbursable nature of many treatments, and multi-facetedness of KM treatment. In order to collect KM data, it is necessary to collect patient information with a purpose, such as establishing a registry, to increase precision and usability. Registry refers to continuously and systematically collected data related to the occurrence of diseases to find out the level of occurrence of the disease in the region or hospital and managing it.^[21]^

This study has great significance. This study is the first registry-based study for collecting real-world data of patients receiving HM for weight loss in primary KM clinics. In South Korea, of the total 15,167 medical institutions affiliated with the KM health industry, KM primary clinics account for approximately 93% with 14,106 institutions. This explains the need of research in primary medical units by observing the real-world data of KM.^[22]^ The result of this registry may show the current status of patients receiving HM treatments for weight loss and provide evidence for reasonable decision-making on KM healthcare policy for obese patients in the future. Second, this study is an intervention-based registry focusing on HM in KM. It has great significance in accurately collecting the prescription composition and dose of HM data for obesity treatment in actual clinical practice on a standardized registry platform. This registry can contribute greatly in providing evidence on the safety of obese patients who have been receiving treatment with HM through large-scale data collection for primary medical institutions. Third, this study will apply the HM AEs template developed through a systematic method by an expert Delphi group. Six types of AEs, including headache, nausea, chest palpitations, dry mouth and thirst, constipation, and insomnia, were statistically determined through the Delphi method (data are not shown) and the terms representing AEs were used according to the Medical Dictionary for Regulatory Activities.^[23]^ It would be helpful to collect systematic AEs of HM for weight loss. Fourth, this registry aims to collect the data of 1000 patients visiting the primary KM clinics. This is the largest KM registry focusing on primary KM clinics. This registry will enroll patients receiving HM for weight loss; thus, a large patient sample can be enrolled to investigate the natural history and clinical characteristics of these patients, including demographics, treatment history and results, and progress.^[24]^ Lastly, this registry data will reflect on the KM clinical practice guidelines for obesity. This study is a part of the project named “Advancement of Korean Medicine Clinical Practice Guideline and Critical pathway for Obesity” that aims to develop and disseminate clinical practice guidelines for obesity in KM.^[25]^ The data will be used to verify hypotheses for KM weight loss treatment, including safety of HM. Moreover, it has been planned to reflect the safety verification results of HM, which are urgently required in the current version of KM clinical practice guidelines,^[26]^ and will contribute greatly to the KM community.

## Author contributions

**Conceptualization:** Mi Mi Ko, Sungha Kim.

**Data curation:** Mi Mi Ko, Bo-Young Kim.

**Methodology:** Mi Mi Ko, Bo-Young Kim, Sungha Kim.

**Resources:** Mi Mi Ko, Bo-Young Kim, Sungha Kim.

**Supervision:** Sungha Kim.

**Writing – original draft:** Mi Mi Ko.

**Writing – review & editing:** Mi Mi Ko, Bo-Young Kim, Mi Ju Son, Kyung Hwan Jegal, Won-Seok Chung, Sungha Kim.
